# Cervical Spine Chordoma With Tracheal and Vascular Invasion: A Case Report

**DOI:** 10.7759/cureus.87937

**Published:** 2025-07-14

**Authors:** Arya Kermanshah, Christine Shen, Max Soghikian

**Affiliations:** 1 Anesthesiology, University of Miami, Miami, USA; 2 Anesthesiology, University of California San Diego, San Diego, USA

**Keywords:** airway obstruction, chordoma, difficult airway, difficult airway algorithm, hemorrhage, interventional radiology guided embolization, palliative care, spine oncology, tracheostomy

## Abstract

Chordomas are rare, locally aggressive tumors from notochordal remnants, often involving critical neurovascular structures. We present a case of a tracheostomy patient with metastatic cervical chordoma causing significant airway obstruction and vascular encasement, leading to failed attempts to modify her existing tracheostomy, recurrent hemorrhage, and ultimately death. We review the literature on airway-compromising chordomas, detail the multidisciplinary management of this patient's airway, and propose considerations for adapting the difficult airway algorithm in cases involving critical airway compromise. This case highlights the challenges of managing advanced disease and the need for evolving airway management protocols tailored to patients with known critical airways.

## Introduction

Chordomas are rare, slow-growing malignant tumors that originate from remnants of the embryonic notochord, typically located along the axial skeleton [1,2]. These tumors are most commonly found in the sacrum (50%), skull base (35%), and mobile spine, particularly in the cervical vertebrae (15%) [3]. The literature provides several examples highlighting the rarity of such cases. For instance, oropharyngeal chordomas are exceedingly rare, with only 11 cases reported as of 2017 [4]. Similarly, nasopharyngeal chordomas are also uncommon, as described in a case report where a nasopharyngeal chordoma was incidentally diagnosed during a diagnostic examination for sleep-disordered breathing [5]. Another study reviewed five cases of extraosseous chordomas involving the nasopharynx, emphasizing their rarity and the diagnostic challenges they present [6].

Cervical chordomas can present with airway obstruction or dysphagia due to local invasion. Although no specific incidence of airway invasion is reported, National Comprehensive Cancer Network (NCCN) guidelines and case series have documented cervical tumors extending into the oropharynx and retropharyngeal space, impacting airway patency [7,8].

Chordomas are classified as low- to intermediate-grade sarcomas but demonstrate local aggressiveness, frequent recurrence, and a five-year survival rate of ~50% [9]. Prognosis is shaped by tumor location, extent of resection, and dedifferentiation, with worse outcomes associated with upper cervical location, intralesional margins, and absence of radiotherapy [10].

Management involves en bloc surgical resection when feasible, followed by high-dose radiation therapy, often utilizing proton beam or carbon-ion radiotherapy. Despite aggressive therapy, recurrence and metastasis, most commonly to the lung and bone, are frequent [11]. Cervical chordomas pose airway management challenges due to their proximity to the trachea and esophagus. Anterior surgical approaches can lead to complications like dysphagia and aspiration. In a study by Yu et al., 36.4% of patients required postoperative swallow studies, and 27% needed tracheostomies, some long-term, underscoring the need for meticulous perioperative planning [12].

Tracheostomy management is essential in advanced chordomas of the skull base or cervical spine, where airway compromise is common. In one cohort, 31% of patients required prolonged postoperative airway support, with risk factors including cranial nerve involvement, tumor size, and surgical approach [13]. Mechanical complications, particularly tube displacement or obstruction, are frequent and potentially life-threatening. The Royal College of Anaesthetists' 4th National Audit Project identified displacement as the leading cause of critical tracheostomy events, especially in obese or mobile patients [9]. In response, the National Tracheostomy Safety Project established widely adopted guidelines emphasizing early detection with waveform capnography and prioritizing oxygenation over ventilation [14].

Additionally, bleeding around the tracheostomy site, whether from vessels damaged during insertion or from rare complications like trachea-innominate artery fistulas, can lead to life-threatening situations. Immediate actions to control bleeding, such as suction, compression, or the use of hemostatic agents, are essential, and surgical consultation is often required [15]. Surgical emphysema, resulting from tracheostomy tube displacement, can also occur, and forceful ventilation should be avoided to prevent further complications. Lastly, cuff management of tracheostomy tubes is critical, with regular pressure checks to prevent tracheal injury, stenosis, or aspiration [15].

Collaboration between anesthesia, interventional radiology, and critical care teams is essential for effective airway management and optimizing patient outcomes, given the complexity of tracheostomy care in patients with chordomas. Although the difficult airway algorithm is well-established, the perioperative airway management for patients with these critical airways can be more nuanced. Here, we present a case involving multidisciplinary care for a chordoma patient who required emergent surgical intervention for hemorrhage around her critical tracheostomy site.

This report complies with the Health Insurance Portability and Accountability Act (HIPAA); HIPAA authorization for publication was obtained from the decedent's next of kin.

## Case presentation

A 59-year-old woman with metastatic cervical chordoma and a prior tracheostomy presented with significant hemorrhage from the oropharynx and nasopharynx as well as bleeding around her tracheostomy site, with the majority of the blood originating above the tracheostomy. This event occurred one week after a month-long hospitalization for progressive dyspnea due to tumor compression of the trachea.

Oncologic history

**Figure 1:**
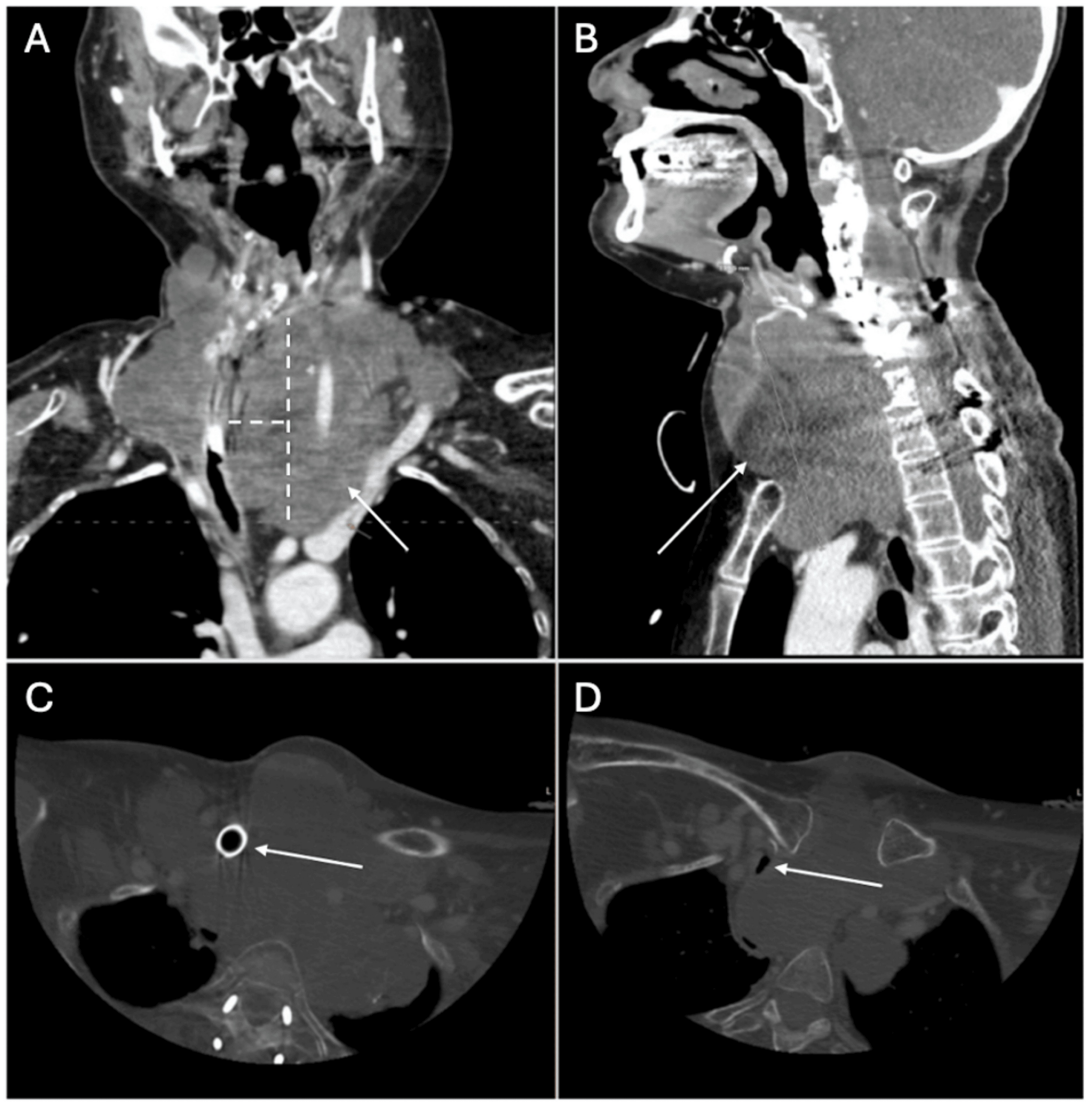
Head and neck CTA Sagittal (A), Coronal (B): High attenuation fluid is noted in the pharynx, suggesting the presence of blood. Similar appearance of the transspatial chordoma throughout the cervical spine and neck compared to prior imaging with chronic bilateral carotid and vertebral artery encasement, severe rightward deviation of the airway, and effacement of the esophagus. The tracheostomy tube courses through the mass with persistent narrowing of the airway distal to the tracheostomy tube. CT axial views (C, D): Tracheostomy tube visualized (C). Airway patency at the next slice distal to the tracheostomy tube diameter of 7 mm (D). CTA: computed tomography angiography

Due to the onset of right-sided Horner's syndrome, she was worked up and diagnosed in 2006. She underwent surgery and radiation, followed by proton beam therapy and neck dissection in 2012 for recurrence. Her disease remained stable until 2016, when prednisone was started for dyspnea. In 2018, tracheostomy and PEG (percutaneous endoscopic gastrostomy) tube placement became necessary due to chronic hypoxic respiratory failure. A 2019 magnetic resonance angiography (MRA) showed encasement of the bilateral carotid and vertebral arteries with tracheal displacement. By 2024, imaging revealed a stable tumor size but worsening tracheal narrowing (7×4 mm), pharyngeal deviation, encasement of the subclavian and carotids, and a new brachiocephalic vein thrombus. Computed tomography angiography (CTA) also showed pulmonary metastases without embolism (Figure 1).

Initial airway management

During her hospitalization, the interventional pulmonary team determined that the existing Tracoe 7-mm uncuffed tracheostomy tube (74 mm length) could not bypass the stenosis. A 110-mm tube, long enough to bypass the lesion, was unavailable commercially. Due to high procedural risk, airway instrumentation was deferred. She was started on a steroid taper and discharged for palliative planning.

Re-presentation, airway evaluation, and initial interventions

She returned one week later with worsening dyspnea and bleeding near the tracheostomy. Emergency department vitals included heart rate 145 bpm, respiratory rate 32 breaths/minute, SpO₂ 98%, blood pressure 136/116 mmHg, and temperature 37.4 °C. Given her history and active bleeding, carotid blowout syndrome was suspected.

Bedside tracheoscopy did not reveal an active source of bleeding, and visualization was limited by the presence of blood and tumor obstruction but revealed a mass compressing the airway 3 cm distal to the trach. Laryngoscopy could not be safely performed due to the risk of provoking further hemorrhage. In this context, cross-sectional imaging was necessary to assess for pseudoaneurysm, arterial extravasation, or other vascular abnormalities that might require urgent endovascular intervention. A CTA of the head and neck was therefore obtained to rapidly evaluate for vascular injury prior to committing to angiography or surgical exploration.

Neuro-interventional radiology performed a CTA that ruled out a pseudoaneurysm or active bleeding, followed by empirical particle embolization of the right and left thyrocervical arteries. However, despite embolization of the bilateral thyrocervical arteries and administration of tranexamic acid, the patient continued to experience recurrent oropharyngeal hemorrhages.

Multidisciplinary airway planning

**Figure 2:**
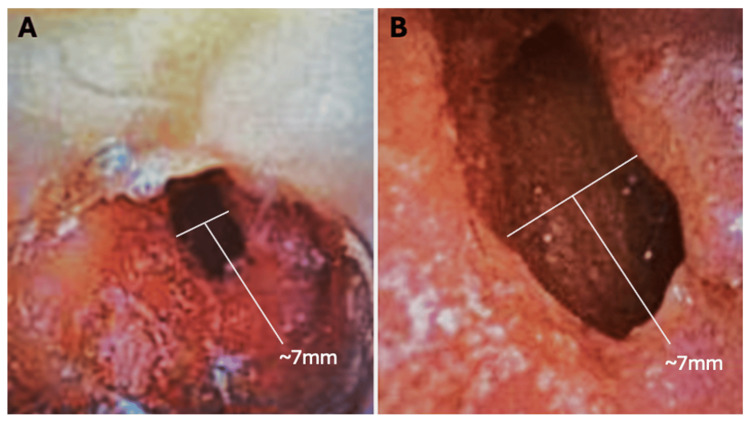
Tracheoscopy view showing tumor invasion and severe tracheal narrowing Optimal fiberoptic views of the airway through the tracheostomy tube, demonstrating a critically narrowed, slit-like residual lumen with near-complete obstruction (A, B). Bright reflections correspond to mucosal surfaces illuminated by the scope's light source.

This case hinged on high-stakes multidisciplinary decision-making due to the patient's tenuous airway anatomy and catastrophic bleeding risk. Given the large, infiltrative cervical chordoma, it was clear that if the existing tracheostomy were dislodged, the tumor would collapse into the tracheal defect, making re-establishment of the airway impossible and resulting in rapid death. Extensive preoperative discussions with anesthesia, ENT, neurointerventional radiology, and critical care centered on the likelihood of airway loss and the patient's goals of care in that scenario. The team agreed that induction could only occur after confirming effective ventilation and tidal volumes through the existing trach, which had been sealed off by tumor. Airway visualization was limited, requiring blind passage of a small ETT (endotracheal tube) through the trach and confirmation with auscultation and pressure monitoring. A fiberoptic scope was used through the trach to estimate carina distance, and an airway exchange plan using an Aintree catheter (Cook Medical, Bloomington, IN, USA) was prepared as a last resort. These collaborative steps allowed for induction with minimized risk of catastrophic airway failure and were pursued only after the patient and family understood the high risk of intraoperative mortality, summarized here:

We attached the anesthesia circuit to her existing tracheostomy and found that we could not achieve adequate tidal volumes. To guide our tube placement, we passed a fiberoptic scope through the trach to estimate the distance to the carina, since the extra-long trach meant the ETT tip needed to sit above the carina with room to inflate the cuff. Although ultra-thin bronchoscopes that fit a 4.5-mm ETT are commercially available, one was not on hand; we passed the tube "blind" (Figure 2) through the trach without resistance, inflated its cuff, and immediately achieved good tidal volumes on the ventilator. Once adequate ventilation and oxygenation were confirmed by arterial blood gas (ABG) analysis, we induced anesthesia and proceeded without respiratory issues. As a precaution, an Aintree catheter exchange set was primed and ready in case re-intubation or tube exchange became necessary.

Outcome

The patient was transferred to the ICU postoperatively with ongoing bleeding from the oral cavity, bilateral nares, and tracheostomy site. Despite topical hemostatic measures initiated by ENT, including oral cavity packing, liberal application of Afrin, and topical tranexamic acid, bleeding persisted. By postoperative day one, she experienced a sudden hemodynamic collapse around 2 p.m. despite escalating vasopressors and transfusion support. Neurointerventional radiology was consulted, but no culprit vessel was identified on angiography, and further embolization was deemed unsafe due to the risk of compromising critical CNS perfusion. They recommended against empiric embolization and advised continued medical management with tranexamic acid and ongoing goals of care discussions. As head swelling progressed, likely due to venous outflow obstruction and baroreceptor compression, neurologic decline became apparent, and maximum-dose vasopressors failed to stabilize her. In collaboration with neurosurgery and ENT, and following a multidisciplinary meeting with the family, care was transitioned to a comfort-focused approach. The patient passed away that evening, surrounded by loved ones. This case highlights the complexities involved in managing a critical tracheostomy and airway crisis in the setting of invasive, recurrent chordoma and the importance of perioperative considerations in maintaining a secure airway.

## Discussion

This case shows the serious health problems caused by invasive cervical chordoma, which can block the airway and damage blood vessels. Although chordomas are generally slow-growing, their locally aggressive nature leads to substantial difficulty in achieving complete surgical resection in some cases, as these tumors often invade surrounding critical structures, such as the trachea and major vessels [1,2]. In this patient, the tumor's progression involved challenges associated with advanced chordoma, including vascular encasement, airway compromise, and recurrent hemorrhage.

Airway management challenges

Airway management in patients with chordomas is particularly difficult due to both intrinsic tracheal narrowing from tumor invasion and extrinsic compression by the surrounding mass. Current guidelines for difficult airway management typically focus on failed intubation scenarios rather than tumor-induced airway obstruction [16]. In fact, up to 31% of patients with skull base chordomas require prolonged airway support, a factor that correlates with increased pulmonary complications [17]. This emphasizes the importance of a nuanced approach to airway management, where proactive, early interventions are essential in preventing life-threatening complications.

The American Society of Anesthesiologists (ASA) Difficult Airway Algorithm provides a flexible, decision-tree-based tool that considers risks such as ventilation difficulty, aspiration, and desaturation when selecting an airway management pathway, including awake intubation. However, it lacks specific guidance for anatomically fixed or progressively narrowing airways, such as those caused by locally aggressive tumors. This omission limits its utility in cases like ours, where traditional anatomical access points may be obscured or distorted [18].

Recent reviews emphasize that oncology patients often present with unique anatomical and physiological predictors of airway difficulty, from tumor mass effect to post-radiation fibrosis. These features may fall outside conventional prediction tools. Innovations such as non-invasive oxygenation strategies, extracorporeal membrane oxygenation (ECMO), and multidisciplinary airway teams are increasingly necessary in this population, suggesting that airway management protocols should evolve to address the distinct needs of this population [19]. Even after the widespread dissemination of guidelines such as the ASA Difficult Airway Algorithm, studies like the UK NAP4 and subsequent reviews highlight persistent challenges in the real-world management of acutely obstructed airways, especially those complicated by tumor burden. These cases often require deviation from standard algorithms in favor of individualized strategies tailored to tumor location, patient physiology, and available resources [20].

One of the key challenges in this case was managing the airway obstruction. It required careful decision-making about whether awake management of the airway was feasible, which involved consulting with the ENT team and evaluating the potential risks and benefits of such an approach. The use of a fiberoptic scope allowed for visualization of the airway, ensuring that the patient could be maintained in a stable state while plans for further management were discussed.

Approach to the difficult airway

To safely manage airway induction, the team first confirmed poor tidal volumes with the anesthesia circuit attached to the existing tracheostomy. A fiberoptic scope was used to estimate the distance to the carina. Lacking an ultra-thin scope, a 4.5-mm cuffed ETT was blindly advanced through the trach, with ventilation confirmed via auscultation, capnography, and ABG. Once adequate oxygenation was assured, anesthesia was induced. An Aintree catheter exchange set was prepared as a contingency for tube failure or dislodgement.

End-of-life considerations

Despite embolization of the bilateral thyrocervical arteries and medical management with tranexamic acid, the patient continued to experience recurrent hemorrhages, resulting in hemodynamic instability and ultimately death. This outcome underscores the terminal trajectory that some recurrent chordomas can take, particularly when involving critical airway and vascular structures. It also emphasizes the importance of integrating palliative care early in the management process, especially in patients with tumors that cause significant anatomical disruption.

## Conclusions

Despite a structured difficult airway algorithm, multidisciplinary collaboration, and endovascular interventions, the tumor's anatomical invasion into the trachea and surrounding vasculature made it inoperable and ultimately fatal. As advances in imaging, radiation therapy, and systemic treatments prolong survival, more clinicians will face the downstream complexities of recurrent or metastatic chordomas invading vital airway structures. Early, proactive airway evaluation is essential in this population, particularly when anatomical compromise is expected to progress.

This case underscores the importance of tailoring airway protocols to tumor-related obstruction, including developing preemptive rescue strategies and integrating palliative goals early in the decision-making process. Secure tracheostomy management, multidisciplinary planning, and flexibility beyond standard algorithms are all vital for navigating the airway risks presented by locally aggressive chordomas. Ultimately, sustaining airway patency in such patients may be the most crucial determinant of both safety and dignity at the end of life.
